# Genome-Wide Identification and Characterization of the PPO Gene Family in Cotton (*Gossypium*) and Their Expression Variations Responding to *Verticillium* Wilt Infection

**DOI:** 10.3390/genes14020477

**Published:** 2023-02-13

**Authors:** Shuhan Yang, Qun Ge, Sumei Wan, Zhihao Sun, Yu Chen, Yanfang Li, Qiankun Liu, Juwu Gong, Xianghui Xiao, Quanwei Lu, Yuzhen Shi, Renhai Peng, Haihong Shang, Guodong Chen, Pengtao Li

**Affiliations:** 1 College of Agriculture, Tarim University, Alar 843300, China; 2 School of Biotechnology and Food Engineering, Anyang Institute of Technology, Anyang 455000, China; 3 State Key Laboratory of Cotton Biology, Key Laboratory of Biological and Genetic Breeding of Cotton, The Ministry of Agriculture, Institute of Cotton Research, Chinese Academy of Agricultural Sciences, Anyang 455000, China

**Keywords:** *Gossypium*, PPO gene family, bioinformatics analysis, qRT-PCR, *Verticillium* wilt

## Abstract

Polyphenol oxidases (PPOs) are copper-binding metalloproteinases encoded by nuclear genes, ubiquitously existing in the plastids of microorganisms, plants, and animals. As one of the important defense enzymes, PPOs have been reported to participate in the resistant processes that respond to diseases and insect pests in multiple plant species. However, PPO gene identification and characterization in cotton and their expression patterns under *Verticillium* wilt (VW) treatment have not been clearly studied. In this study, 7, 8, 14, and 16 PPO genes were separately identified from *Gossypium arboreum*, *G. raimondii*, *G. hirsutum*, and *G. barbadense*, respectively, which were distributed within 23 chromosomes, though mainly gathered in chromosome 6. The phylogenetic tree manifested that all the PPOs from four cotton species and 14 other plants were divided into seven groups, and the analyses of the conserved motifs and nucleotide sequences showed highly similar characteristics of the gene structure and domains in the cotton PPO genes. The dramatically expressed differences were observed among the different organs at various stages of growth and development or under the diverse stresses referred to in the published RNA-seq data. Quantitative real-time PCR (qRT-PCR) experiments were also performed on the GhPPO genes in the roots, stems, and leaves of VW-resistant MBI8255 and VW-susceptible CCRI36 infected with *Verticillium dahliae* V991, proving the strong correlation between PPO activity and VW resistance. A comprehensive analysis conducted on cotton PPO genes contributes to the screening of the candidate genes for subsequent biological function studies, which is also of great significance for the in-depth understanding of the molecular genetic basis of cotton resistance to VW.

## 1. Introduction

Polyphenol oxidases (PPOs) are copper-binding metalloproteinases encoded by nuclear genes, which ubiquitously exist in the plastids of microorganisms, plants, and animals. As one of the earliest enzymes to be studied, PPO was first found to be related to the sap that was hardening in Venn wood in 1883, and it was subsequently extracted from mushrooms in 1938 [1]. PPOs are capable of catalyzing the oxidation of monophenol hydroxyl and bisphenol hydroxyl into diphenol and resorcinol, which could be broadly divided into three categories: monophenol oxidase (MPO, tyrosinase, EC.1.14.18.1), diphenol oxidate (DPO, catecholase, EC.1.10.3.2), and laccase (EC.1.10.3.1) [2]. Among the three PPO types, DPO is mainly distributed in plants, while most MPO and laccase are observed in microorganisms [3]. The functions of PPO proteins in plants are largely determined by three conserved domains, namely, KFDV, tyrosinase, and DWL, which have been reported not only to cause enzymatic browning of vegetables, fruits, teas, and other agricultural products [4], but also to participate in plant response to biotic and abiotic stresses [5]. Furthermore, PPOs were found to be related to plant resistance against pathogenicity, which could form a protective layer for protecting cells from bacteria by catalyzing the formation of lignin and flavone compounds and directly came into play in disease tolerance by forming flavonoids [6]. In consideration of their potentially significant functions in plants, it is necessary to conduct systematic analyses of the biological information and expressing patterns responding to adversity stress in a specific cash crop.

To date, the PPO gene family has been identified and characterized in multiple plants, for which the relative information and evidence were obtained by PCR amplification of genomic DNA or, originally, from the BAC library [7,8]. In recent years, PPO genes have been gradually subjected to systematic analyses of the bioinformatics on the published genomes [9,10,11]. With the rapid development of high-throughput sequencing technology, more and more complete genomes of model and non-model organisms have been successfully sequenced. These genomes not only provided adequate and accurate reference information for subsequent function research, but they also laid solid foundations for the deep comprehension of polyploid properties [12]. Cotton is one of the most significant economic crops globally, of which the natural fibers, plant proteins, edible oil, and other agricultural by-products greatly contribute to China’s economic progress [13]. However, *Verticillium* wilt caused by *V. dahliae* is currently a major problem in cotton production, and this soil-borne fungal disease hinders the normal development of the cotton plant, finally affecting the cotton yield and fiber quality [14,15]. *V. dahliae* is harbored in quite a wide host range (over 600 kinds of plants) and possesses a strong survival ability and a constantly mutating pathogenicity [16]. These characteristics not only drove the development of VW to be the dominant toxicity type gradually, but they also made it difficult to prevent and control. Moreover, there was the lack of a suitable bactericide for *V. dahliae*, and all the factors eventually led to failures in crop rotation, chemical fumigation, and soil amendments in cotton production [17]. Therefore, the most effective and economical prevention and control measure at present remains to select and promote cotton varieties with high VW resistance. Previous studies showed that either polyphenolic content or PPO activity was observed to correlate with VW infection [18,19], while there were few reports on the function of the PPO protein family in the adversity stresses of cotton. In this study, PPO proteins were firstly identified from cotton genomes with different polyploid species, which were subsequently subjected to bioinformatic analysis and a qRT-PCR experiment. All the results provided a theoretical basis for studying the characteristics and functions of PPO proteins in cotton when responding to VW infection.

## 2. Materials and Methods

### 2.1. Identification of PPO Genes

To systematically explore PPO family genes in plants, genome-wide identification was conducted on four cotton species and six other plant genera by a comprehensive bioinformatics procedure. The selected cotton genomes consisted of two diploid ones, *G. arboreum* (ftp://bioinfo.ayit.edu.cn/downloads/ (accessed on 7 May 2018)) [20] and *G. raimondii* (https://phytozome.jgi.doe.gov/pz/portal.html#!info?alias=Org_Graimondii (accessed on 20 December 2012)) [21], and two tetraploid ones, *G. hirsutum* and *G. barbadense* (http://ibi.zju.edu.cn/cotton/ (accessed on 18 March 2019)) [22]. The other plants included *Glycine max* [23], *Zea mays* [24], *Medicago truncatula* [25], *Oryza sativa* [26], *Theobroma cacao* [27], and *Populus trichocarpa* [28], of which the genome data were obtained from the JGI database (http://www.phytozome.net (accessed on 1 January 2012)) and NCBI database (https://www.ncbi.nlm.nih.gov/ (accessed on 30 July 2018)). Due to the conserved PPO domains of PPO1_DWL (pfam12142), Tyrosinase (pfam00264), and PPO1_KFDV (pfam12143), the corresponding HMM (Hidden Markov Model) profiles were downloaded from the Pfam database (http://pfam.xfam.org/ (accessed on 8 January 2019)) [29]. BLAST (Basic Local Alignment Search Tool) with parameters of score value ≥ 0.0001 and E-value = 1 × 10^−3^ and HMMER3.1 (http://www.hmmer.org/ (accessed on 20 October 2011)) [30] with the default parameters were utilized to determine the candidate sequences. Subsequently, the obtained putative PPO genes were submitted to the National Center for Biotechnology Information Conserved Domain database (CDD, https://www.ncbi.nlm.nih.gov/Structure/cdd/wrpsb.cgi (accessed on 1 January 2011)) [31] and SMART database (http://smart.embl-heidelberg.de/ (accessed on 1 January 2009)) [32] for further confirmation. Meanwhile, the properties of PPO family genes in cotton were also analyzed by the online tools of softberry PROT_MAP (http://linux1.softberry.com/berry.phtml?topic=prot_map&group=programs&subgroup=xmap (accessed on 1 July 2012)), softberry ProtComp (http://linux1.softberry.com/berry.phtml?topic=protcompan&group=programs&subgroup=proloc (accessed on 1 July 2012)), and ExPASy (https://web.expasy.org/translate/ (accessed on 1 July 2012)) [33]. The parameters of PPO properties mainly included mRNA length, CDS (coding sequences) length amino acid length, molecular weight (MW), and theoretical isoelectric point (pI).

### 2.2. Phylogenetic Analysis and Classification of PPO Genes

In order to construct the phylogenetic tree of all the PPO genes in plants, sequence alignment was firstly performed by multiple alignment using fast Fourier transform (MAFFT Melbourne Australia, v7.407) with default parameters and L-INS algorithms. Based on the results of conserved site sequences screened by software Gblock (http://molevol.cmima.csic.es/castresana/Gblocks_server.html, 0.91b (accessed on 1 August 2007)) [34], a phylogenetic tree was constructed by IQ-TREE (http://www.iqtree.org/, v.1.6.9 (accessed on 3 November 2015)) [35] with the maximum likelihood. The substitution model was calculated with ModelFinder (intergraded in IQ-TREE; best-fit model: JTT + R5, chosen according to BIC), and the resulting treefile was visualized by R/ggtree [36] and AI (Adobe Illustrator CS6) [37].

### 2.3. Analysis of PPO Gene Structure and Protein Domain in Cotton

Structure information of PPO genes was firstly acquired from the annotation files of *G. arboreum*, *G. raimondii*, *G. hirsutum*, and *G. barbadense*, which were subsequently loaded into Gene Structure Display Server (GSDS, http://gsds.cbi.pku.edu.cn/ (accessed on 15 August 2007)) [38] for visualization analysis on the exon/intron structure of PPO genes. Meanwhile, protein sequences of cotton PPO genes were loaded into the online program Pfam (http://pfam.xfam.org/ (accessed on 23 June 2020)) to analyze the conserved domain of candidate genes, and TBtools (https://github.com/CJ-Chen/TBtools) [39] software was also utilized for visual analysis of protein domain of cotton PPOs.

Due to being the most widely planted and the most productive cotton species in the world, *G. hirsutum* attracted increasing research on how to improve fiber yield and quality. Therefore, it is of great significance to screen the relative candidate genes for controlling these important agronomic traits, as well as their corresponding regulatory elements. A set of transcriptome data conducted on *G. hirsutum* cultivar CCRI36 and its chromosome segment substitution line (CSSL) offspring MBI8255 by our group was also utilized for verification of expression patterns of PPO genes and investigation on potential regulatory elements. Hence, the upstream region of 2000 bp started from the translation initiation site, which was generally regarded as the eukaryotic promoter, of which the genome sequence was extracted from the *G. hirsutum* PPO genes for prediction of cis-acting elements. PlantCARE (http://bioinformatics.psb.ugent.be/webtools/plantcare/html/ (accessed on 1 January 2002)) [40] was utilized in this study to online analyze the promoter sequence of GhPPO genes, and all the cis-acting elements related with the hormone and adversity stress were identified. TBtools was used for visual analysis of GhPPO cis-acting components.

### 2.4. Expression Pattern Analysis of PPO Genes in Cotton

For determining the expression profiles of PPO genes in the four cotton species, the raw data of transcriptome sequencing were downloaded from Nation Center of Biotechnology Information (NCBI, https://www.ncbi.nlm.nih.gov/ (accessed on 30 July 2018)). The expression reads of *G. arboreum* cultivar Shixiya1 were generated by performing RNA-seq on the eleven different organs (root, stem, leaf, bract, cotyledon, petal, phloem, anther, stigma, sepal, and seed) and developing ovules (0, 5, 10, and 20 DPA, days post anthesis), which were obtained from the SRA databse (PRJNA594268) [41]. RNA-seq was conducted on the two organs (leaf and stem), the developing seeds (10, 20, 30, and 40 DPA), the developing ovules (0 and 3 DPA), and the developing fibers (10 and 20 DPA) of *G. raimondii*, which were retrieved from the SRA databse (PRJNA171262) [21]. The expression reads of *G. hirsutum* accession TM-1 and *G. barbadense* cultivar Hai7124 were obtained from the SRA databse (PRJNA490626), and their same RNA-seq samples included the ten different organs (root, filament, bract, torus, anther, pental, stem, sepal, leaf, and pistil), the developing ovules (−3, −1, 0, 1, 3, 5, 10, 20, and 25 DPA), and the developing fibers (10, 20, and 25 DPA) [22]. Different adversity stresses were also conducted on the RNA-seq samples of the two tetraploid cotton species, including NaCl, PEG, 4 °C, and 37 °C, which were collected at 1, 3, 6, 12, and 24 h. These RNA-seq data were subjected to standardization processing and quantization of expression levels by utilizing lg (TPM + 1, transcripts per kilobase of exon model per million mapped reads). Based on the TPM values of PPO genes, R/pheatmap (https://cran.r-project.org/web/packages/pheatmap/index.html (accessed on 13 April 2020)) [42] was chosen to generate heatmap.

### 2.5. RNA Extraction and Gene Expression Analysis by Real-Time Quantitative PCR

*G. hirsutum* variety CCRI36 (stably susceptible to VW) and its chromosome segment substitution line (CSSL) offspring MBI8255 (stably resistant to VW) were planted in disposable paper cups filled with mixed media of vermiculite and sand (6:4 *v*/*v*), which were placed in an incubator in the laboratory of Anyang Institute of Technology (Anyang, Henan province) in 2022. When two cotyledons were open and flat, the seedlings were firstly pulled out and infected with 1 × 10^7^ spores/mL *V. dahliae* fungus V991 (virulent and deciduous strain) via root-dipping method. The infected plants were subsequently planted back in the disposable paper cups after inoculation for 1 minute [43]. Then, samples of roots, stems, and leaves at 0, 1, and 2 days after inoculation (DAI), to be infected with *V. dahliae* V991, were separately collected, which were utilized for RNA extraction by FastPure Universal Plant Total RNA Isolation Kit (Product code: RC411-01, Vazyme, Nanjing, China). RNA concentration and integrity were observed on NanoDrop 2000 spectrophotometer (Thermo scientific, Waltham, MA, USA), with quality that was then detected by 2% agarose gel electrophoresis. Subsequently, HiScript Ⅲ All-in-one RT SuperMix Perfect for qPCR (Product code: R333-01, Vazyme, Nanjing, China) was adopted to deal with the high-quality RNA, and the reversely transcriptional cDNA was stored in the refrigerator at −20 °C. The specific primers of GhPPOs were designed according to their CDS sequences of *G. hirsutum* genome on the primer-blast of NCBI website; meanwhile, the constitutive-expression *GhUBQ7* was chosen as the reference gene [44]. Real-time PCR experiment was conducted on ABI7500 Fast system (Applied Biosystems, Waltham, MA, USA) with total reaction volume of 20 μL, including 2 × ChamQ Universal SYBR qPCR Master Mix 10 μL, 10 mmol/L forward primer 0.4 µL, 10 mmol/L reverse primer 0.4 µL, cDNA template 2 µL, and ddH_2_O 7.2 µL, and their amplification conditions were set as follows: 95 °C, 60 s; (95 °C, 10 s; 60 °C, 30 s) × 40 cycles; 95 °C, 15 s; 60 °C, 60 s, 95 °C, 30 s; 60 °C, 15 s. Three biological replicates were designed together with three technical replicates in qRT-PCR experiments, and −2^ΔΔct^ algorithm was adopted to calculate the relative expression levels [45].

## 3. Results

### 3.1. Identification and Characteristics of PPO Genes in Gossypium

Having conducted a genome-wide analysis of four cotton species, we identified 7, 8, 14, and 16 PPO genes in *G. arboreum*, *G. raimondii, G. hirsutum*, and *G. barbadense*, respectively. Meanwhile, there were 17 PPOs in *G. max*, four PPOs in *Z. mays*, four PPOs in *M. truncatula*, two PPOs in *O. sativa*, and seven PPOs in *T. cacao*. The results of analyses on the nucleic acid sequences of cotton PPO genes showed that the coding regions were 1242–1848 bp (encoding 413–615 amino acids) in *G. arboreum*, 1647–1860 bp (encoding 548–619 amino acids) in *G. raimondii*, 1647–1884 bp (encoding 548–627 amino acids) in *G. hirsutum*, and 939–2277 bp (encoding 312–758 amino acids) in *G. barbadense*. In addition, the physiochemical characteristics of the cotton PPO proteins were analyzed, with protein lengths and molecular weights that ranged from 312 aa to 758 aa and 35.39 kDa to 86.70 kDa, respectively. The isoelectric points of the cotton PPOs ranged from 5.29 to 7.83, with an average value of 6.84, indicating their amino acidic preference (Table 1).

### 3.2. Chromosome Distribution of PPO Gene Family in Cotton

**Figure 1:**
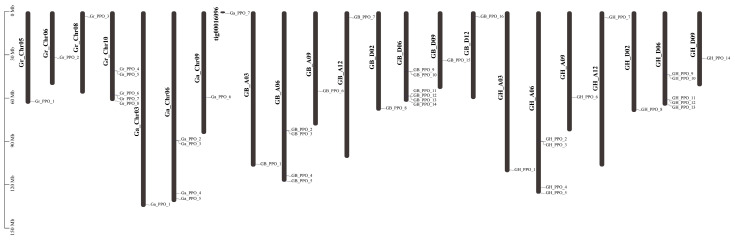
Chromosomal localization analysis of cotton PPO gene members. *Y*-axis indicates the chromosome length (bp).

Based on the annotation files of the published cotton genomes, the chromosome distributions of the cotton PPO genes were subjected to visualization analysis, which produced a physical location map (Figure 1). In the diploid genome *G. arboareum*, seven GaPPO genes were distributed on three chromosomes and one scaffold, namely, Chr03, Chr06, Chr09, and tig00016096, respectively, of which the most genes were located in Chr06. As for the other diploid genome, *G. raimondii*, only four chromosomes were observed for eight GrPPO genes, namely, Chr05, Chr06, Chr08, and Chr10, and the most genes were located in Chr10. In the tetraploid genome *G. hirsutum*, 14 GhPPO were distributed on seven chromosomes, including seven A-subgnomic and seven D-subgnomic genes. As for the other tetraploid genome, *G. barbadense*, 16 GbPPO genes were distributed on eight chromosomes, including seven A-subgnomic and nine D-subgnomic genes. Moreover, similar results were observed for the same seven chromosomes, namely, ChrA03, ChrA06, ChrA09, ChrA12, ChrD02, ChrD06, and ChrD09, while different results were observed for the newly found ChrD12 and another two GbPPO genes distributed in ChrD06 and ChrD12. Except for only one PPO gene that was distributed in ChrA03, ChrA09, ChrA12, ChrD02, ChrD09, and ChrD12, more PPO genes were clustered in ChrA06 and ChrD06, especially in ChrD06 (five GhPPO and six GbPPO genes), indicating the potential importance of chromosome 6 for the PPO genes in cotton. 

### 3.3. Structure Analysis of PPO Gene Family

**Figure 2:**
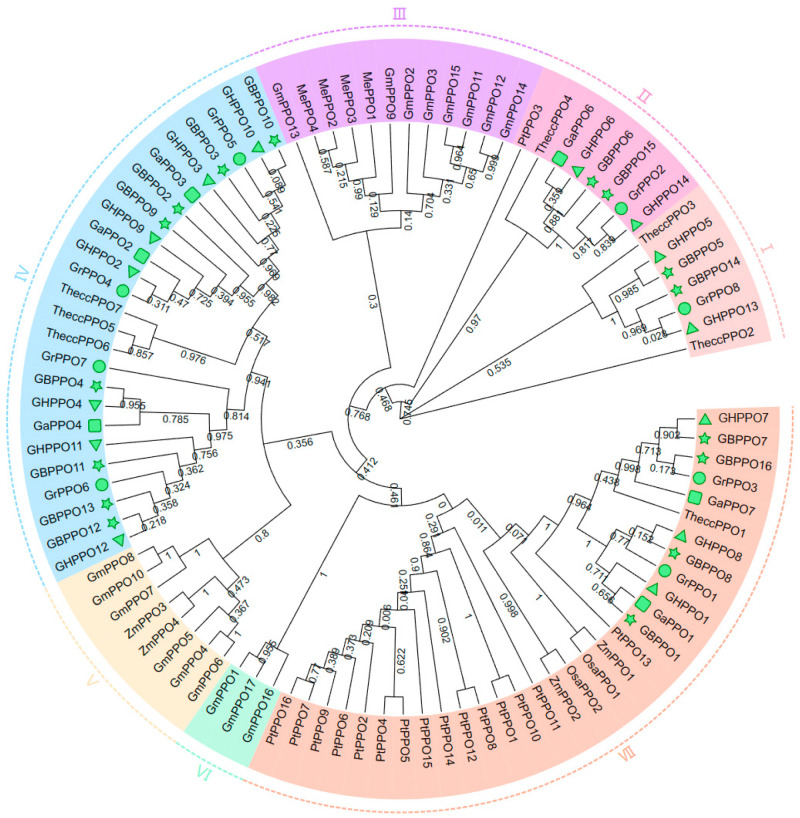
Phylogenetic tree of PPO genes among *G. arboreum* (GaPPO), *G. raimondii* (GrPPO), *G. hirsutum* (GhPPO), *G. barbadense* (GbPPO), *G. max* (GmPPO), *P. trichocarpa* (PtPPO), *O. sativa* (OsaPPO), *Z. mays* (ZmPPO), *M. truncatula* (MePPO), and *T. cacao* (TheccPPO).

The phylogenetic tree was constructed to synthetically investigate the evolutionary relationships of PPO genes (Figure 2), and all the PPO proteins in plants could be divided into seven groups (Ⅰ–Ⅶ). Among the groups, the highest numbers of PPO genes were observed in Group Ⅴ and Group Ⅶ, and their total number, 54 members, accounted for 59.57% of all the PPO proteins. The cotton PPO proteins were distributed in Group Ⅰ, Group Ⅱ, Group Ⅴ, and Group Ⅶ, of which 20 PPOs (44.44% of the total 45 cotton PPO proteins) were clustered in Group Ⅴ, together with three *T. cacao* PPOs. Despite that, there were other plant PPOs gathered into the four groups related with cotton PPOs, where only *T. cacao* PPOs were always presented, indicating a possibly closer relationship between cotton and *T. cacao*。

### 3.4. Analyses of Gene Structure and Protein Domain of Cotton PPOs

**Figure 3:**
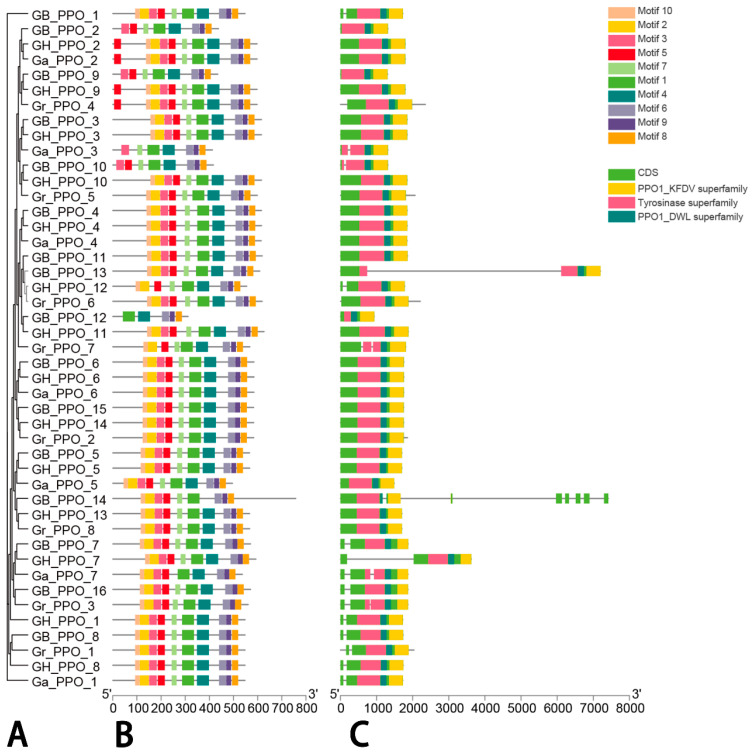
Analyses of evolutionary relationship, conserved motif, and gene structure of PPO family in cotton. Note: (**A**) represents the evolutionary tree of cotton PPO genes, (**B**) represents the conserved motif of cotton PPO genes, and (**C**) represents the gene structure of exon and intron of cotton PPOs. The scale axis of (**B**,**C**) indicate the sequence length.

In general, diverse combinations of exons and introns as well as their differences in number might result in multiple functions among the genes, which motivated us to perform analyses of the gene structures and protein domains of cotton PPOs (Figure 3). Most of the homologous PPO genes from the different cotton genomes presented one-to-one corresponding relationships located in the same chromosomes, such as among the diploid AA-genome (*G. arboreum*) and the tetraploid subgenome of *G. hirsutum*-AA and *G. barbadense*-AA or the diploid DD-genome (*G. arboreum*) and the tetraploid subgenome of *G. hirsutum*-DD and *G. barbadense*-DD (Figure 3A). However, inconsistent phenomena were observed between the ChrD06-located *GrPPO2* and the ChrD09-located *GbPPO15* and *GhPPO14*; between the ChrD10-located *GrPPO8* and the ChrD06-located *GbPPO14* and *GhPPO13*; between the ChrD12-located *GbPPO16* and the ChrD08-located *GrPPO3*; and between the ChrD02-located *GbPPO8* and the ChrD05-located *GrPPO1*.

Gene structure was also investigated for all the cotton PPO genes (Figure 3C). The results showed that their coding sequences contained non-conserved and conserved domains, including PPO1_DWL (pfam12142), tyrosinase (pfam00264), and PPO1_KFDV (pfam12143). However, their non-coding sequences consisted of different numbers of introns. The number of introns ranged from zero to seven: there were no introns in 28 PPOs, one intron in 13 PPOs, two introns in 3 PPOs, and seven introns in 1 PPO, implying that most PPO genes have similar structures with the characteristic of aggregation (Supplementary Materials, Table S1).

Meanwhile, similar motif models were observed in the whole PPO genes of the cotton species, and a total of 10 conserved motifs were identified by the PlantCARE tool, except for five motifs of *GbPPO12*, seven motifs of *GaPPO3*, and eight motifs of *GbPPO2*, *GbPPO9*, and *GbPPO10* (Figure 3B and Table S1). In order to further illustrate the potential relationship between cotton PPO genes and specific biological processes, a 2.0 kb promoter region was extracted to screen the cis-acting elements, and, in total, 301 cis-acting elements were obtained from the GhPPO genes, including two typical core ones, namely, TATA and CAAT (Figure S1). Using statistics, 15 kinds of cis-acting elements were identified, which were involved in the possible functions, such as stress response, hormone regulation, cell development, MYB binding, and metabolic control. Among the stress-response elements, five types in the GhPPO promoters consisted of anaerobic, drought, low temperature, defense, and pressure response elements. In addition, abscisic acid (ABA), gibberellin (GA), auxin (IAA), methyl jasmonate (MeJA), and salicylic acid (SA) were also observed in the PPO promoter region of *G. hirsutum*, all of which are present in the GhPPO family, while the whole members responded to at least one hormone. Another two cis-acting elements related to cell development were found to participate in the expressions of endosperm and meristem, which were separately presented in six and three GhPPO genes, respectively. The cis-element of circadian rhythm relevant to circadian rhythm regulation was found in four GhPPO genes, and many light-responsive elements were also observed in the GhPPO promoter. These cis-acting elements identified from the GhPPO promoter region implied potentially important functionalities in specific biological processes, such as growth and development, stress responses, and metabolic control.

### 3.5. Expression Analysis of PPO Gene Family in Diploid Cotton

**Figure 4:**
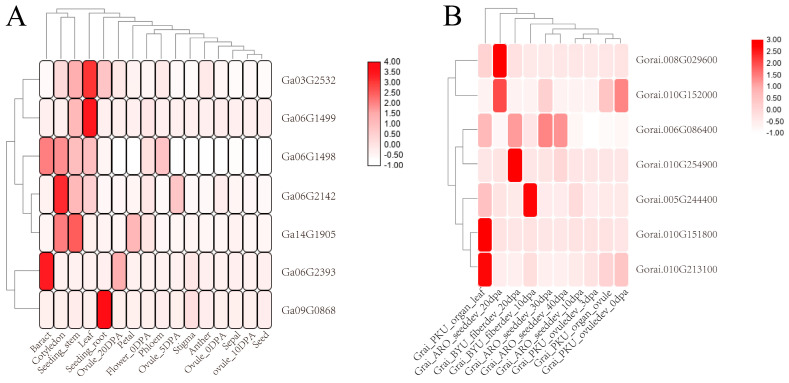
Expression heatmap of PPO genes of the diploid cotton species in different organs at the diverse stages of development. Note: (**A**) represents the expression situation of PPO genes of *G. arboreum* in different organs at the diverse stages of development, and (**B**) represents the expression situation of PPO genes of *G. raimondii* in different organs at the diverse stages of development.

By comparing the expression levels of the PPO family genes of *G. arboreum* in different organs at diverse stages of development (Figure 4A), we found that *GaPPO1* (*Ga03G2532*) was highly expressed in the cotyledon, seedling stem, and leaf, and *GaPPO3* (*Ga06G1499*) was only highly expressed in the cotyledon and seedling stem. Higher expression levels of *GaPPO2* (*Ga06G1498*) were observed in the bract, cotyledon, seedling stem, leaf, and phloem, which were also found in the cotyledon, seedling stem, leaf, and 5 DPA-ovule of *GaPPO4* (*Ga06G2142*). *GaPPO7* (*Ga14G1905*) was highly expressed in the seedling stem, leaf, and petal, while *GaPPO5* (*Ga06G2393*) was highly expressed in the bract and ovule 20 DPA (days post anthesis). The relatively high expression level of *GaPPO6* (*Ga09G0868*) was only found in the seedling root, indicating that most GaPPO genes have higher expression levels in specific tissues of seedlings.

The expression patterns of the PPO family genes of *G. raimondii* were also analyzed in different tissues under diverse periods (Figure 4B). *GrPPO3* (*Gorai.008G029600*) was highly expressed in the seeds of 20 DPA, and *GrPPO5* (*Gorai.010G152000*) showed higher expression levels in the seeds of 20 DPA and the ovule of 0 DPA. The expression level of *GrPPO2* (*Gorai.006G086400*) in the leaves and fibers of 20 DPA and the seeds of 30 DPA and 40 DPA was higher than that in other tissues, while the similar result occurred in the fibers of 20 DPA in *GrPPO8* (*Gorai.010G254900*) and of 10 DPA in *GrPPO1* (*Gorai.005G244400*). The unique tissue with a higher expression level of *GrPPO4* (*Gorai.010G151800*) and *GrPPO6* (*Gorai.010G213100*) was the leaf, implying that there are dramatically diverse expression patterns in different tissues.

**Figure 5:**
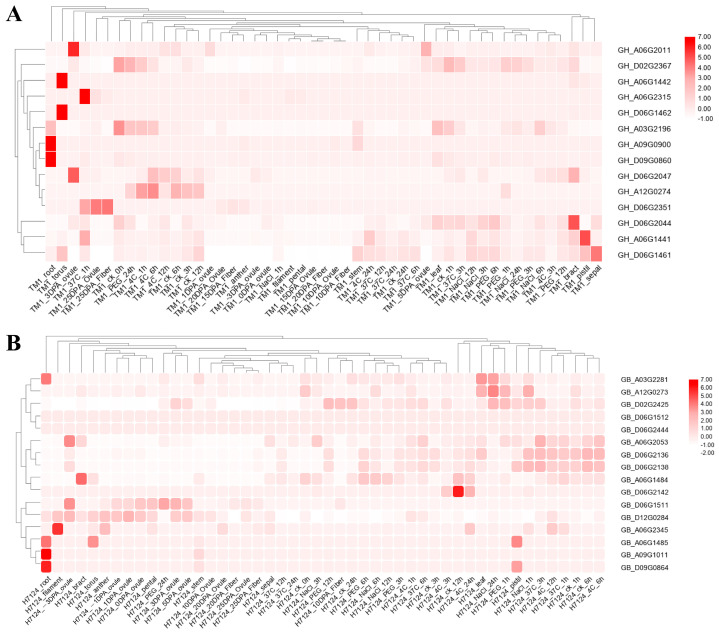
Expression heatmap of PPO genes of the tetraploid cotton species in different organs at the diverse stages of development or under various stress treatments. Note: (**A**) represents the expression situation of PPO genes of *G. hirsutum* in different organs at the diverse stages of development or under various stress treatments, and (**B**) represents the expression situation of PPO genes of *G. barbadense* in different organs at the diverse stages of development or under various stress treatments.

Likewise, the tetraploid cotton species was chosen to perform an expression-level analysis in different tissues under diverse periods and stress treatments. As shown in Figure 5A for *G. hirsutum* (TM-1), we found that *GhPPO4* (*GH_A06G2011*) was highly expressed in the ovules of 3 DPA and 5 DPA, while *GhPPO8* (*GH_D02G2367*) had higher expression levels under normal growth or control check (ck) for 0 h and 1 h; meanwhile, there were increased expression levels in response to PEG treatment for 24 h. The unique tissue with higher expression levels was the torus in *GhPPO3* (*GH_A06G1442*) and *GhPPO10* (*GH_D06G1462*), and a similar phenomenon was also observed in response to 37 °C treatment for 1 h of *GhPPO5* (*GH_A06G2315*) and in the roots of *GhPPO6* (*GH_A09G0900*) and *GhPPO14* (*GH_D09G0860*). *GhPPO1* (*GH_A03G2196*) was highly expressed both in the leaf and under ck for 0 h, and *GhPPO12* (*GH_D06G2047*) was highly expressed not only in the ovules of 3 DPA and the bracts but also under 4 °C treatment for 6 h. A higher expression level of *GhPPO7* (*GH_A12G0274*) was observed both under 4 °C treatment for 1 h and 6 h and under ck for 6 h, while *GhPPO13* (*GH_D06G2351*) was highly expressed in the ovule and fiber of 25 DPA and under 37 °C treatment for 1 h. *GhPPO11* (*GH_D06G2044*) had higher expression levels in the leaves and bracts and under PEG treatment for 6 h, and *GhPPO2* (*GH_A06G1441*) was highly expressed in the pistil and under 37 °C treatment for 1 h and 4 °C treatment for 24 h. *GhPPO9* (*GH_D06G1461*) was highly expressed in the sepal, stem, and torus.

Based on the expression situations of PPO genes for *G.barbadense* (Hai7124) in different tissues and under diverse stresses (Figure 5B), we found that *GbPPO1* (*GB_A03G2281*) was highly expressed in the roots and leaves and under NaCl treatment for 24 h, while *GbPPO7* (*GB_A12G0273*) had a higher expression level in the leaves, under NaCl treatment for 1 h and 24 h, and under PEG treatment for 1 h. *GbPPO8* (*GB_D02G2425*) was highly expressed not only in the fiber of 10 DPA but also under PEG treatment for 1 h and 12 h and under NaCl treatment for 24 h and ck; only *GbPPO10* (*GB_D06G1512*) and *GbPPO14* (*GB_D06G2444*) showed a few expression levels in all their tissues or under the different stresses. *GbPPO4* (*GB_A06G2053*) was highly expressed in the ovules of 3 DPA and under 37 °C treatment for 3 h, while *GbPPO11* (*GB_D06G2136*) was highly expressed under NaCl treatment for 1 h and 3 h and under 4 °C treatment together with ck treatment for 6 h. Higher expression levels of *GbPPO12* (*GB_D06G2138*) were observed in the pistil, under NaCl treatment for 1 h, under 4 °C treatment for 3 h and 6 h, and under ck treatment for 6 h, while *GbPPO2* (*GB_A06G1484*) was highly expressed in the bracts and under ck treatment for 12 h. *GbPPO13* (*GB_D06G2142*) had a higher expression level under ck treatment for 12 h and under 4 °C treatment for 24 h, while *GbPPO9* (*GB_D06G1511*) was highly expressed in the ovules of −3 DPA and under PEG treatment for 24 h. The only tissues with a higher expression level were the ovule of 1 DPA for *GbPPO16* (*GB_D12G0284*) and the roots of *GbPPO6* (*GB_A09G1011*), though similar tissues were also observed in the fiber and anther of *GbPPO5* (*GB_A06G2345*), in the roots and pistil of GbPPO15 (GB_D09G0864), and in the roots, torus, and pistil of *GbPPO3* (*GB_A06G1485*). Combined with the expression patterns of the above-mentioned GhPPO and GbPPO genes, we concluded that the tetraploid PPOs were not only highly expressed in different tissues but also responded to PEG, NaCl, cold, and hot treatments.

### 3.6. Expression Investigation of GhPPO Genes in Response to VW Infection

**Figure 6:**
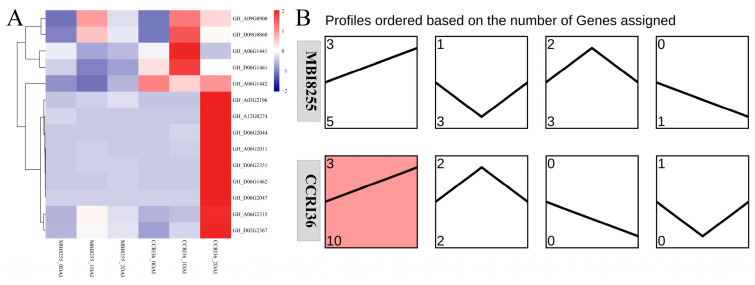
Expression heatmap and patterns of GhPPO genes in CCRI36 and MBI8255 roots infected by V991 for 0, 1, and 2 DAI. Note: (**A**) represents the expression heatmap of GhPPOs in CCRI36 and MBI8255 roots infected by V991 for 0, 1, and 2 DAI, and (**B**) represents the temporal expression of GhPPOs in CCRI36 and MBI8255 roots infected by V991 for 0, 1, and 2 DAI.

In consideration of the positive correlation between PPO activity and *Verticiliium* wilt infection deduced by our previous study [43], the corresponding transcriptome data were subjected to a re-mapping procedure into the TM-1 genome [22], which aimed at investigating the expression variations of the GhPPO genes responding to *V. dahliae* V991 inoculation. According to the heatmap analysis of the PPO family genes in VW-resistant MBI8255 and VW-susceptible CCRI36 infected with V991 at 0, 1, and 2 DAI (days after inoculation), all the GhPPOs dramatically showed various expression patterns, of which most had higher expression levels in CCRI36 than those in MBI8255 under the same V991-infection period (Figure 6A). The Short Time-series Expression Miner (STEM) was also chosen to conduct temporal expression analysis on the 14 GhPPO genes, resulting in four kinds of profiles with different expression patterns gathered by 12 PPO genes (Figure 6B). The highest number of PPO genes presented a continually upregulated trend (profile 3) in both MBI8255 and CCRI36, among which only 10 PPO genes were significantly enriched in CCRI36. The other two PPO genes in CCRI36, namely, *GhPPO2* (*GH_A06G1441*) and *GhPPO14* (*GH_D09G0860*), were clustered into profile 2, presenting an upregulated trend and then a downregulated trend, while the same profile in MBI8255 included *GhPPO14* (*GH_D09G0860*), *GhPPO4* (*GH_A06G2011*), and *GhPPO6* (*GH_A09G0900*). For the PPO genes clustered into profile 0 and profile 1 in CCRI36, the former presented a continually downregulated trend for *GhPPO7* (*GH_A12G0274*), while the latter showed a downregulated and then upregulated trend for *GhPPO2* (*GH_A06G1441*), *GhPPO3* (*GH_A06G1442*), and *GhPPO11* (*GH_D06G2044*).

**Figure 7:**
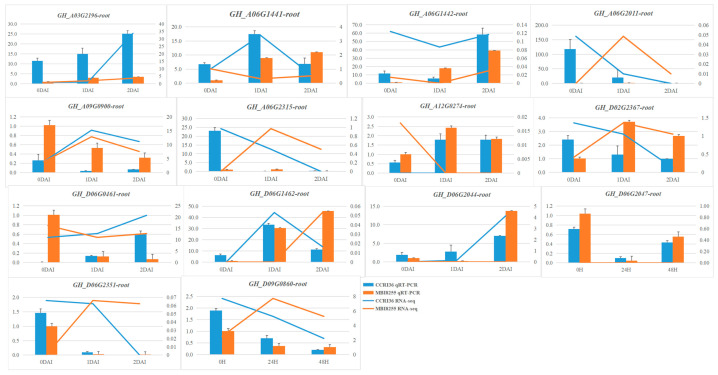
qRT-PCR verification of GhPPO genes in CCRI36 and MBI8255 roots infected by V991 for 0, 1, and 2 DAI.

Real-time quantification was also conducted on the 14 GhPPO genes in the CCRI36 and MBI8255 roots, under the same situation as the transcriptome experiment (Figure 7), for which the qRT-PCR results of 10 GhPPOs showed a high consistency with the RNA-seq data, indicating the reliability of the dramatic changes of the PPO genes when responding to VW infection. Meanwhile, the plant stems and leaves of the CCRI36 and MBI8255 infected with V991 were subjected to qRT-PCR experiments (Supplementary Materials, Figures S2 and S3), and we found that higher expression levels were mostly observed in the VW-susceptible tissues compared to those in the VW-resistant tissues at the same stages of V991 infection. The results signified more downregulated PPOs in response to cotton VW, which was consistent with the corresponding qRT-PCR and RNA-seq results of the cotton roots.

## 4. Discussion

As specific copper metal enzymes, PPO proteins widely exist in plant species [46]. An increasing number of studies have demonstrated that PPOs are of great significant not only for plant growth and development, but also for the important roles they play in plant response to biotic and abiotic stresses [47,48]. With the rapid development of genomic sequencing technology, more and more PPO genes have been identified with multiple functions in different plants, for which genome-wide analyses were also conducted in tomato, potato, wheat, poplar, and black cottonwood [49,50,51]. However, genome-wide identification and characterization of the PPO gene family have not yet been fully analyzed in cotton up until now.

In this study, a total of 45 family members of the PPO genes were identified from four cotton species, including 14 PPOs in *G. hirsutum*, 16 PPOs in *G.barbadense*, seven PPOs in *G. arboreum*, and eight PPOs in *G. raimondii*. Among the above-mentioned cotton species, *G. arboreum* (AA) and *G. raimondii* (DD) are diploid cotton species, while *G. hirsutum* and *G.barbadense* are tetraploid cotton species (AADD). It is generally recognized that diploid cotton species are subjected to interspecific hybridization and chromosome reduplication to cultivate tetraploid cotton species [22]. Therefore, the number of PPO genes identified in the tetraploid cotton species was supposed to be the additive summation of the number of PPO genes in the diploid cotton species. We identified the same numbers of PPO genes in the diploid AA-genome (*G. arboreum*) and in the tetraploid AA-subgenomes of *G. hirsutum* and *G.barbadense*, indicating their highly conservative property during the long-term evolution process. There were eight PPO genes identified in the diploid DD-genome (*G. raimondii*), while one less (seven GhPPOs) and one more (nine GbPPOs) were separately observed in the tetraploid DD-subgenomes of *G. hirsutum* and *G.barbadense*, respectively. The different numbers of PPO genes in the DD-genome and DD-subgenomes implied a certain variability of cotton generation. All the PPO genes identified in cotton were unevenly distributed on 23 chromosomes, half of which were located on chromosome 6 (Figure 1), suggesting these changes caused by nonequilibrium might occur before species differentiation.

Based on the phylogenetic tree constructed by the ten plants and four cotton species, all the PPO genes were divided into seven groups (Figure 2). Most of the cotton PPOs were found in groups Ⅰ, Ⅱ, Ⅴ, and Ⅶ, where the largest number of *T. cacao* PPOs were also observed, implying that there is a close evolutionary relationship between *Gossypium* and *Theobroma* [52]. Meanwhile, the gene structure and domain were analyzed for further investigation on the conservative property of the PPO family; thus, 10 conserved motifs (Figure 3B) and three conserved domains (KFDV, tyrosinase, and DWL) (Figure 3C) were successfully screened, and these similarities might confer similar biological functions. There were no introns found in 28 cotton PPO genes (62.22% of the 45 cotton PPOs), one intron found in 13 cotton PPO genes (28.89%), two introns found in three cotton PPO genes (6.67%), and seven introns found in only one cotton PPO gene (2.22%) (Figure 3C). Introns were reported to play an important role in transcriptome regulation [53], which contributes to plants being able to more quickly stimulate gene expression in response to adversity stresses. PPOs could become an important part of oxidative stress reactions, which might result from gene structures with few or no introns [54].

In many plants, the PPO genes were found to respond rapidly to biotic and abiotic stresses [55]. Aiming to seek out some evidence in the promoter region, a number of stress-response cis-elements were found in the GhPPO genes, including anaerobic-response elements, drought-response elements, low-temperature-stress elements, defense elements, and stress-response elements (Figure S1). Several plant-hormone-related elements were also identified in the GhPPO promoter, suggesting that the GhPPO gene family may be involved in the plant-hormone signal-transduction pathways. Specifically, we noticed that some motifs are associated with the ABA, GA, IAA, MeJA, and SA reactions, of which the first one was reported to accumulates under stress conditions, indicating that the ABA-related PPOs might play important roles in the stress response and tolerance of plants and could coordinate reactive oxygen species (ROS) signal-transduction pathways [56].

Despite the expression pattern of the PPO genes in various tissues being confirmed in many species [57,58,59], there was no uniform characteristic of the expression profile of the PPO genes in plants, due to the difference in the PPO numbers of different species. A similar situation occurred in the expression patterns of the cotton PPO genes, and the dramatic differences indicated that these genes may participate in different biological processes or play a role in different biological functions. According to the published RNAseq data of cotton, some PPO genes were preferentially expressed in the cotyledon and seedling stem of *G. arboreum* (Figure 4A), while some were preferentially expressed in the seed of *G. raimondii* (Figure 4B). As for the tetraploid cotton species, the PPO genes were not just preferentially expressed in the root of *G. hirsutum* and *G. barbadenes,* but they also responded to adversity stresses (Figure 5).

Combined with the previous RNA-seq results and the MeJA elements in the promoter regions, the positive correlation between PPO activity and VW infection was again confirmed by performing analyses of the heatmap and STEM profile (Figure 6) on the GhPPO genes in response to V991 infection. Consistent with the RNA-seq results, the qRT-PCR experiments showed a huge difference in the expression levels of the PPO genes in both VW-resistant and VW-susceptible roots under VW infection (Figure 7), of which more downregulated PPOs were observed than upregulated PPOs. Similar results were also found in the qRT-PCR experiments on GhPPO genes either in the stem or leaf of CCRI36 and MBI8255 in response to V991 infection (Figures S2 and S3), indicating that the downregulated PPO genes might be of great significance for cotton plants against VW infection. In the present work, three downregulated GhPPO genes, namely, *GhPPO4* (*Gh_A06G2011)*, *GhPPO11* (*Gh_D06G2044*)*,* and *GhPPO12* (*Gh_D06G2047*), were also identified as candidate genes in response to VW infection. The expression levels of the three GhPPO genes were severely inhibited, suggesting that they may be involved in different physiological events rather than in host defense. Although numerous studies on the biological roles of PPO genes showed that the protein has anti-disease and microbial-invasion effects, detailed information about its function when it comes to new species remains uncertain. More information about the PPO genes found here is needed, through genetic transform technology, to better understand the role these proteins play in the function of upland cotton.

## 5. Conclusions

In this study, a genome-wide analysis of the PPO family was completed in the cotton species, and 45 PPO genes distributed on 23 chromosomes were divided into seven groups. Then, bioinformatics and qRT-PCR were used to analyze the gene structure, phylogeny, chromosomal localization, conserved structure, homeopathic elements, and expression patterns of the PPO genes in cotton. Finally, we found that PPO gene expression changed significantly in response to V991 infection, indicating that the PPO genes may play important roles in VW resistance. This study is also helpful to further explore the function of the PPO genes in cotton.

## Figures and Tables

**Table 1:** Identification and characteristics information of cotton PPO family members.

Gene Name	Gene ID	Genomics Position	Protein Length (aa)	Molecular Weight (kDa)	Isoelectric Point
*Ga_PPO_1* [41]	Ga03G2532	Chr03:133861218–133862947	548	63.195	7.086
*Ga_PPO_2* [41]	Ga06G1498	Chr06:89406195–89407991	598	67.138	6.79
*Ga_PPO_3* [41]	Ga06G1499	Chr06:89662332–89663647	413	47.373	5.922
*Ga_PPO_4* [41]	Ga06G2142	Chr06:125985836–125987683	615	70.181	7.062
*Ga_PPO_5* [41]	Ga06G2393	Chr06:129881213–129882703	496	56.889	6.209
*Ga_PPO_6* [41]	Ga09G0868	Chr09:59384931–59386688	585	65.256	7.612
*Ga_PPO_7* [41]	Ga14G1905	tig00016096:1234774–1236648	537	61.392	7.48
*Gr_PPO_1* [21]	Gorai.005G244400	Chr05:62477068–62479105	548	63.326	6.866
*Gr_PPO_2* [21]	Gorai.006G086400	Chr06:32091285–32093143	584	65.301	7.215
*Gr_PPO_3* [21]	Gorai.008G029600	Chr08:3520590–3522462	561	64.067	7.818
*Gr_PPO_4* [21]	Gorai.010G151800	Chr10:41097418–41099771	598	67.192	6.652
*Gr_PPO_5* [21]	Gorai.010G152000	Chr10:41182365–41184434	599	67.596	6.879
*Gr_PPO_6* [21]	Gorai.010G213100	Chr10:57917180–57919392	619	70.222	6.986
*Gr_PPO_7* [21]	Gorai.010G213300	Chr10:57928797–57930609	568	63.512	5.472
*Gr_PPO_8* [21]	Gorai.010G254900	Chr10:62004848–62006551	567	64.768	6.617
*GH_PPO_1* [22]	GH_A03G2196	A03:109830718–109832447	548	63.247	7.266
*GH_PPO_2* [22]	GH_A06G1441	A06:89879934–89881730	598	67.18	6.79
*GH_PPO_3* [22]	GH_A06G1442	A06:90147702–90149552	616	69.307	7.186
*GH_PPO_4* [22]	GH_A06G2011	A06:121988167–121990017	616	70.378	7.235
*GH_PPO_5* [22]	GH_A06G2315	A06:125891271–125892974	567	64.513	7.05
*GH_PPO_6* [22]	GH_A09G0900	A09:59466573–59468330	585	65.175	7.827
*GH_PPO_7* [22]	GH_A12G0274	A12:4105742–4109367	593	67.96	7.523
*GH_PPO_8* [22]	GH_D02G2367	D02:68093821–68095562	548	63.293	7.086
*GH_PPO_9* [22]	GH_D06G1461	D06:43843765–43845561	598	67.166	6.519
*GH_PPO_10* [22]	GH_D06G1462	D06:43930778–43932628	616	69.617	7.186
*GH_PPO_11* [22]	GH_D06G2044	D06:61154513–61156396	627	71.531	6.51
*GH_PPO_12* [22]	GH_D06G2047	D06:61198543–61200323	555	63.156	6.329
*GH_PPO_13* [22]	GH_D06G2351	D06:64782818–64784521	567	64.815	6.719
*GH_PPO_14* [22]	GH_D09G0860	D09:32552402–32554156	584	65.439	6.917
*GB_PPO_1* [22]	GB_A03G2281	A03:105823405–105825134	548	63.225	7.081
*GB_PPO_2* [22]	GB_A06G1484	A06:82535557–82536873	438	50.192	5.488
*GB_PPO_3* [22]	GB_A06G1485	A06:82746888–82748738	616	69.378	7.022
*GB_PPO_4* [22]	GB_A06G2053	A06:113959340–113961190	616	70.378	7.235
*GB_PPO_5* [22]	GB_A06G2345	A06:117626828–117628531	567	64.513	7.05
*GB_PPO_6* [22]	GB_A09G1011	A09:55231074–55232831	585	65.237	7.827
*GB_PPO_7* [22]	GB_A12G0273	A12:4077941–4079816	571	65.345	7.224
*GB_PPO_8* [22]	GB_D02G2425	D02:67144871–67146608	548	63.278	6.831
*GB_PPO_9* [22]	GB_D06G1511	D06:41409523–41410830	435	49.822	5.647
*GB_PPO_10* [22]	GB_D06G1512	D06:41500819–41502134	417	47.666	5.79
*GB_PPO_11* [22]	GB_D06G2136	D06:58569149–58571008	619	70.365	7.235
*GB_PPO_12* [22]	GB_D06G2138	D06:58617125–58618063	312	35.385	5.289
*GB_PPO_13* [22]	GB_D06G2142	D06:58653169–58660371	609	69.141	7.156
*GB_PPO_14* [22]	GB_D06G2444	D06:62265335–62272753	758	86.703	5.424
*GB_PPO_15* [22]	GB_D09G0864	D09:33963391–33965145	584	65.334	7.242
*GB_PPO_16* [22]	GB_D12G0284	D12:3557843–3559716	571	65.353	7.39

## Data Availability

Not applicable.

## Supplementary Materials

The following supporting information can be downloaded at: https://www.mdpi.com/article/10.3390/genes14020477/s1, Figure S1: Identification of cis-acting elements of GhPPO genes; Figure S2: Expression heatmap and patterns of GhPPO genes in CCRI36 and MBI8255 stems infected by V991 for 0, 1, and 2 DAI. Figure S3: Expression heatmap and patterns of GhPPO genes in CCRI36 and MBI8255 leaves infected by V991 for 0, 1, and 2 DAI. Table S1: Numbers of introns and conserved motifs of cotton PPO genes.
