# Supplementary material for: Genome-Wide Identification and Characterization of the PPO Gene Family in Cotton (Gossypium) and Their Expression Variations Responding to Verticillium Wilt Infection
Source: Genes (Basel). 2023 Feb 13;14(2):477. doi: 10.3390/genes14020477 (PMC9957175; doi:10.3390/genes14020477)
Supplement: Supplementary file 1 [file genes-14-00477-s001.zip › Table S1.pdf]

Table S1 Numbers of introns and conserved motifs of cotton PPO genes

Gene Name	Gene ID	Intron number	Conserved motif number
Ga_PPO_1	Ga03G2532	1	10
Ga_PPO_2	Ga06G1498	0	11
Ga_PPO_3	Ga06G1499	1	7
Ga_PPO_4	Ga06G2142	0	10
Ga_PPO_5	Ga06G2393	0	10
Ga_PPO_6	Ga09G0868	0	10
Ga_PPO_7	Ga14G1905	2	9
Gr_PPO_1	Gorai.005G244400	1	10
Gr_PPO_2	Gorai.006G086400	0	10
Gr_PPO_3	Gorai.008G029600	2	10
Gr_PPO_4	Gorai.010G151800	0	11
Gr_PPO_5	Gorai.010G152000	0	10
Gr_PPO_6	Gorai.010G213100	0	10
Gr_PPO_7	Gorai.010G213300	2	9
Gr_PPO_8	Gorai.010G254900	0	10
GH_PPO_1	GH_A03G2196	1	10
GH_PPO_2	GH_A06G1441	0	11
GH_PPO_3	GH_A06G1442	0	10
GH_PPO_4	GH_A06G2011	0	10
GH_PPO_5	GH_A06G2315	0	10
GH_PPO_6	GH_A09G0900	0	10
GH_PPO_7	GH_A12G0274	1	10
GH_PPO_8	GH_D02G2367	1	10
GH_PPO_9	GH_D06G1461	0	11
GH_PPO_10	GH_D06G1462	0	10
GH_PPO_11	GH_D06G2044	0	10
GH_PPO_12	GH_D06G2047	1	9
GH_PPO_13	GH_D06G2351	0	10
GH_PPO_14	GH_D09G0860	0	10
GB_PPO_1	GB_A03G2281	1	10
GB_PPO_2	GB_A06G1484	0	8
GB_PPO_3	GB_A06G1485	0	10
GB_PPO_4	GB_A06G2053	0	10
GB_PPO_5	GB_A06G2345	0	10
GB_PPO_6	GB_A09G1011	0	10
GB_PPO_7	GB_A12G0273	1	10
GB_PPO_8	GB_D02G2425	1	10

GB_PPO_9	GB_D06G1511	0	8
GB_PPO_10	GB_D06G1512	1	8
GB_PPO_11	GB_D06G2136	0	10
GB_PPO_12	GB_D06G2138	0	5
GB_PPO_13	GB_D06G2142	1	10
GB_PPO_14	GB_D06G2444	7	9
GB_PPO_15	GB_D09G0864	0	10
GB_PPO_16	GB_D12G0284	1	10
