# Supplementary figures and images for: Genome-Wide Identification and Characterization of the PPO Gene Family in Cotton (Gossypium) and Their Expression Variations Responding to Verticillium Wilt Infection
Source: Genes (Basel). 2023 Feb 13;14(2):477. doi: 10.3390/genes14020477 (PMC9957175; doi:10.3390/genes14020477)

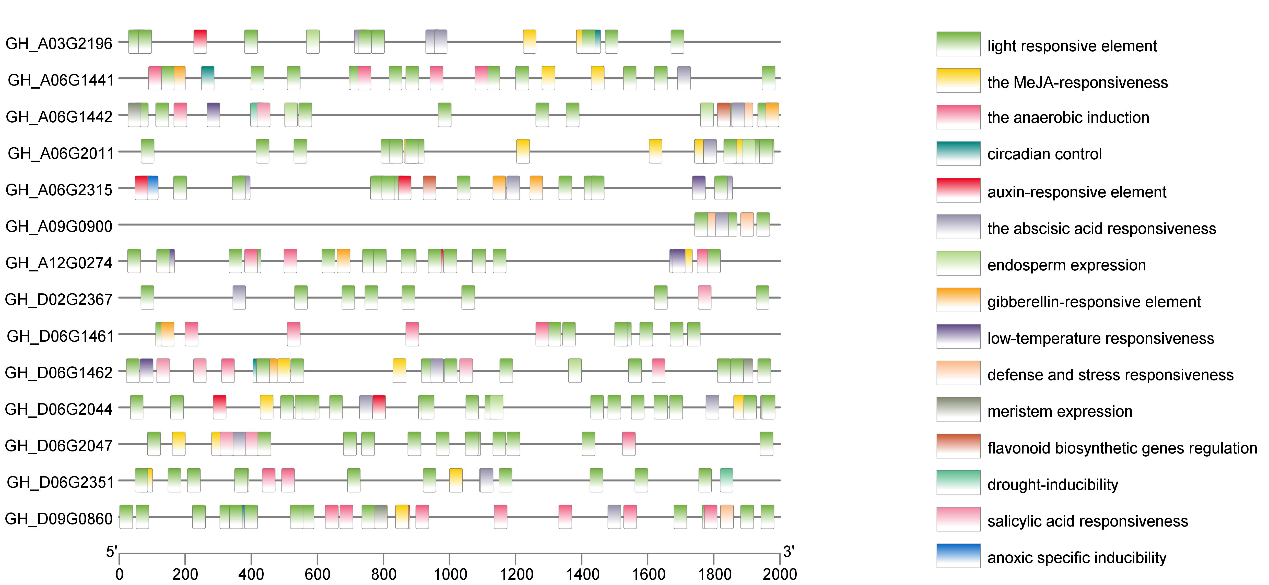

Supplement: Supplementary file 1 [file genes-14-00477-s001.zip › Figure S1.tif]

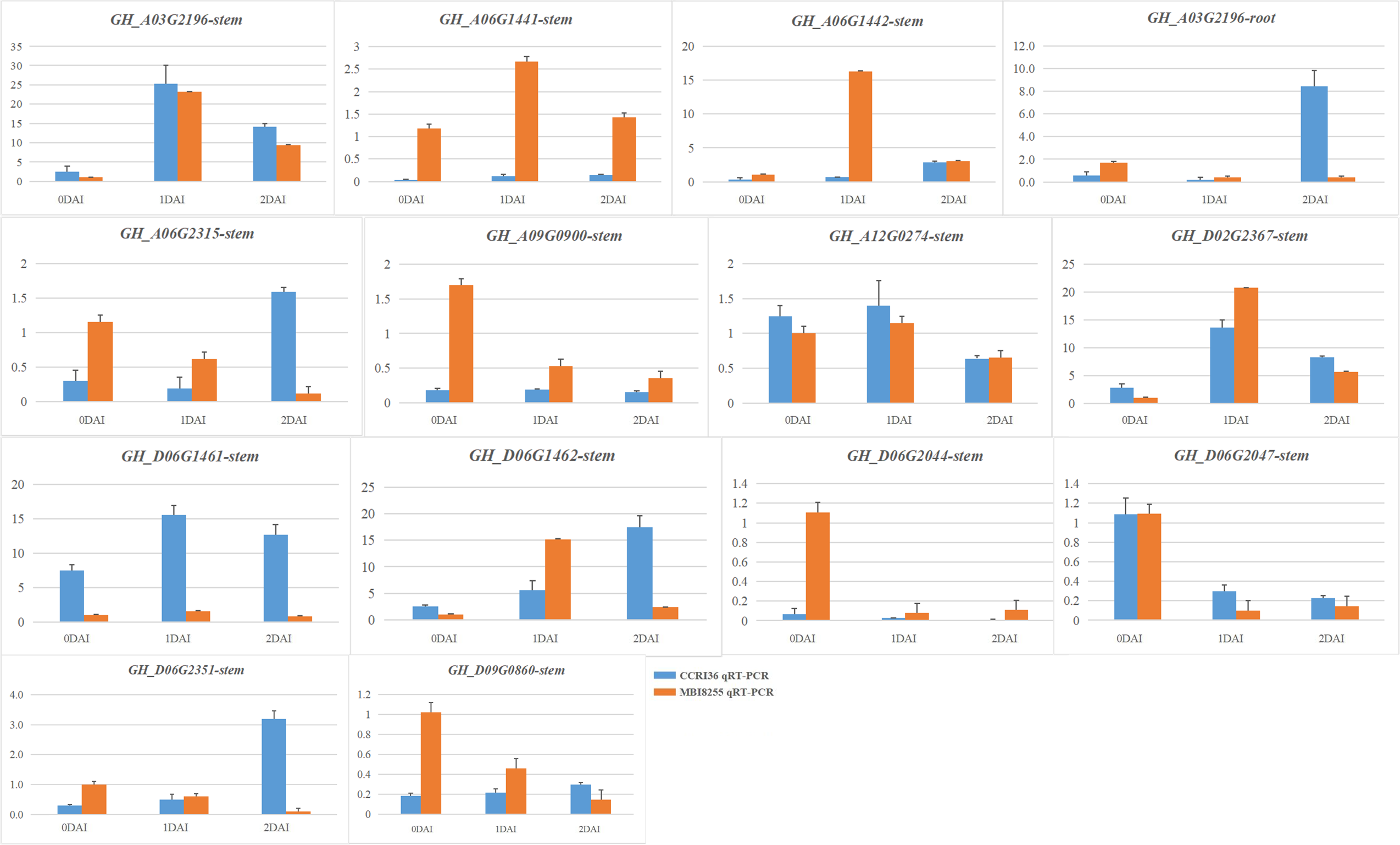

Supplement: Supplementary file 1 [file genes-14-00477-s001.zip › Figure S2.tif]

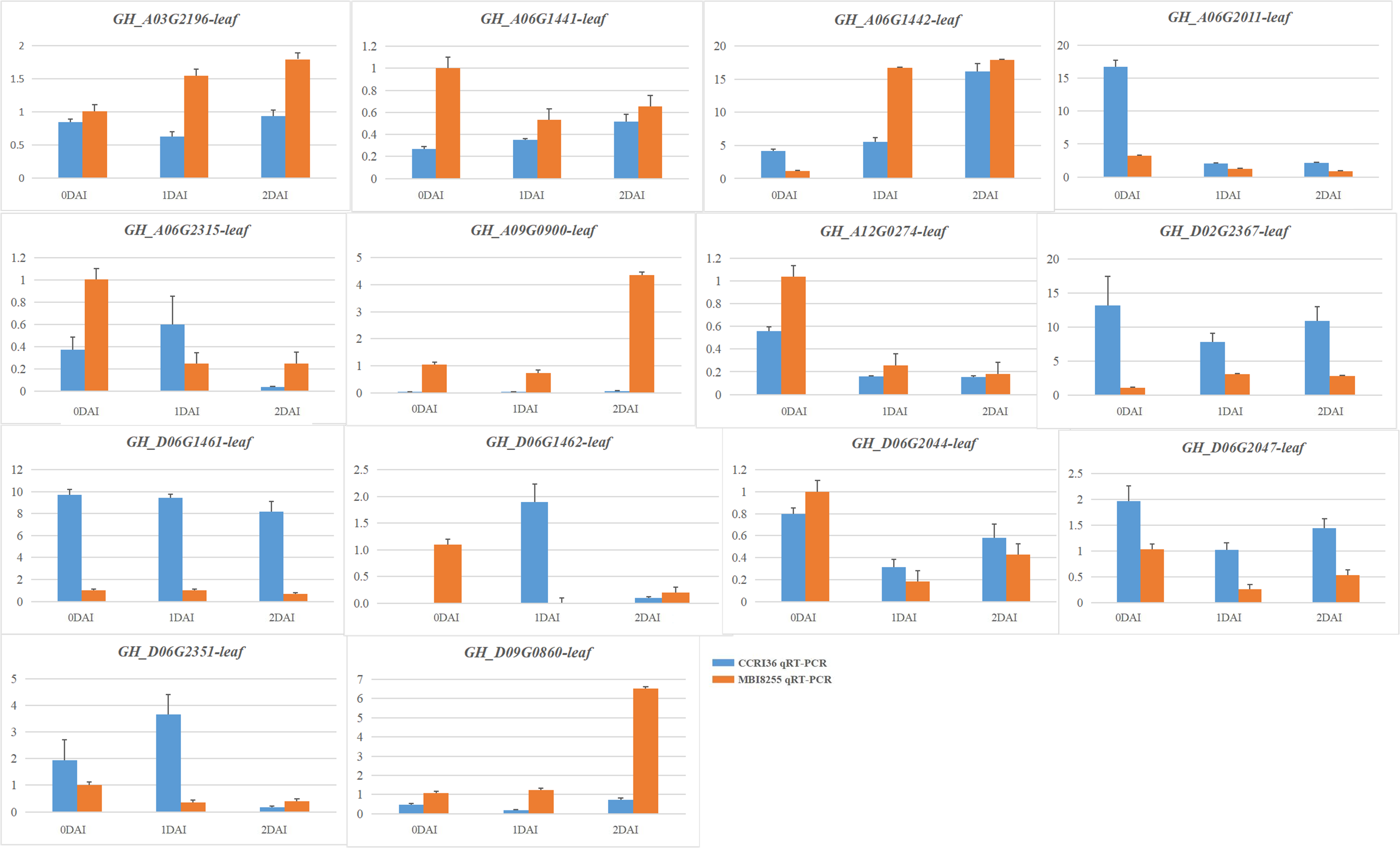

Supplement: Supplementary file 1 [file genes-14-00477-s001.zip › Figure S3.tif]
